# Retrospective data analysis of CLABSI rates at Baystate Medical Center during the COVID-19 pandemic

**DOI:** 10.1017/ash.2023.285

**Published:** 2023-09-29

**Authors:** Giovanni Satta, Kristin Smith, Jacob Smith

## Abstract

**Background:** Central-line–associated bloodstream infections (CLABSIs) are an important public health issue. Recent data from the CDC have shown an increase in healthcare-associated infections (including CLABSI) during the COVID-19 pandemic. Therefore, the main aim of this project was to analyze the epidemiology of central-line–associated bloodstream infection during different periods at the Baystate Medical Center (Springfield, MA) before, during, and after COVID-19 peaks of infection. **Methods:** Two specific periods were considered during the year (quarter January–March and quarter July–September) to consider potential seasonal variations, and the incidence of CLABSI during those 2 quarters was analyzed for 4 different years: 2019 (prepandemic), 2020–2021 (intrapandemic), and 2022 (postpandemic). An analysis of the microbial pathogens causing line infections was also performed to investigate differences described by other authors. **Results:** In total, 97 CLABSI (all from different patients) were reported into the NHSN website during the 8 periods considered. The average age of the patients was 55 years, with a male:female ratio of 57%:43%, and 14 renal patients were on dialysis. The CLABSI rates ranged from a minimum of 1.11 in Q1 of 2020 (start of COVID) to a maximum of 2 in Q3 of 2021 (SARS-CoV-2 delta variant) (Table 1). A statistical comparison of the pre–COVID-19 period with the respective quarters during the pandemic years (2020, 2021, and 2022) did not show any significant differences (Table 2). In term of microbiological data, of the 97 patients with CLABSIs, most of the patients (n = 70) had only 1 pathogen isolated, 14 patients had 2 pathogens, and 3 patients had 3 pathogens, bringing the total number of bacteria cultured to 117. *Candida* spp and *Enterococcus* spp were the most frequently isolated pathogens at 19% and 13%, respectively (Fig. 1). There was no statistically significant difference between the pre–COVID-19 and intra–COVID-19 periods for *Candida* spp (rate ratio, 1.391; 95% CI, 0.5477–3.533; *P* = .48) or *Enterococcus* spp (rate ratio, 2.385; 95% CI, 0.8365–6.798; *P* = .09). **Conclusions:** The COVID-19 pandemic did not seem to have an impact on the local epidemiology at Baystate Medical Center in terms of CLABSI rates or type of pathogens causing infections, but the sample size taken into consideration may not have been powerful enough to detect statistical significance.

**Figure:**
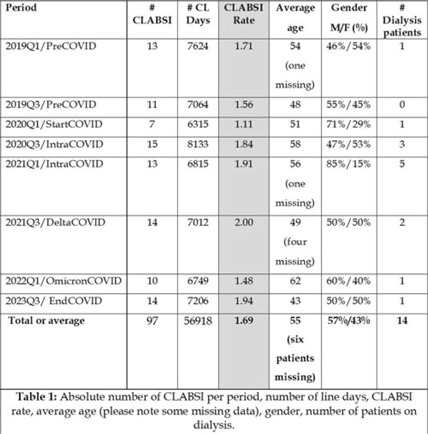

**Figure:**
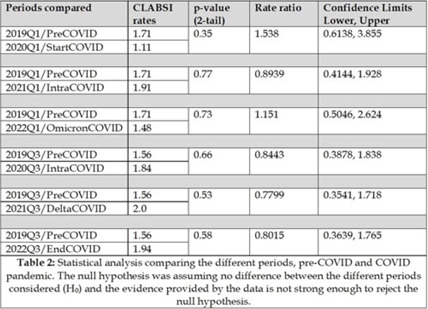

**Figure:**
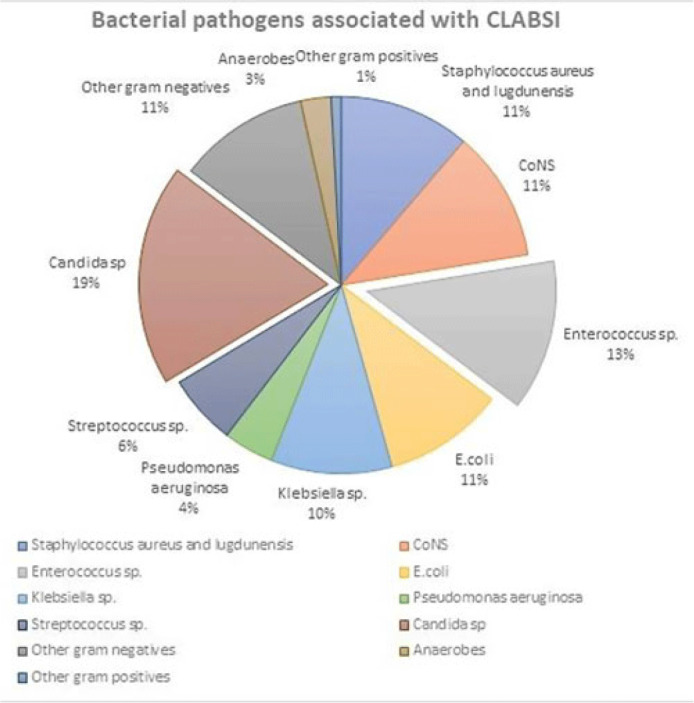

**Note.** This project was carried out as part of Dr Satta’s MPH requirements at UMass.

**Disclosures:** None

